# SLC25A42‐associated mitochondrial encephalomyopathy: Report of additional founder cases and functional characterization of a novel deletion

**DOI:** 10.1002/jmd2.12218

**Published:** 2021-05-04

**Authors:** Mazhor Aldosary, Shahad Baselm, Maha Abdulrahim, Rawan Almass, Maysoon Alsagob, Zainab AlMasseri, Rozeena Huma, Laila AlQuait, Tarfa Al‐Shidi, Eman Al‐Obeid, Albandary AlBakheet, Basma Alahideb, Lujane Alahaidib, Alya Qari, Robert W. Taylor, Dilek Colak, Moeenaldeen D. AlSayed, Namik Kaya

**Affiliations:** ^1^ Department of Genetics King Faisal Specialist Hospital and Research Center Riyadh Saudi Arabia; ^2^ Translational Genomics Department, Center for Genomic Medicine (CGM) King Faisal Specialist Hospital and Research Center Riyadh Saudi Arabia; ^3^ Department of Medical Genetics King Faisal Specialist Hospital and Research Center Riyadh Saudi Arabia; ^4^ King Abdulaziz City for Science and Technology Riyadh Saudi Arabia; ^5^ Wellcome Centre for Mitochondrial Research, Translational and Clinical Research Institute, Newcastle University Newcastle upon Tyne UK; ^6^ NHS Highly Specialised Mitochondrial Diagnostic Laboratory Newcastle upon Tyne Hospitals NHS Foundation Trust Newcastle upon Tyne UK; ^7^ Department of Biostatistics, Epidemiology, and Scientific Computing King Faisal Specialist Hospital and Research Center Riyadh Saudi Arabia; ^8^ College of Medicine Alfaisal University Riyadh Saudi Arabia

**Keywords:** ATP production, deep brain stimulation, mitochondrial oxygen consumption, *SLC25A42*, truncation

## Abstract

SLC25A42 is the main transporter of coenzyme A (CoA) into mitochondria. To date, 15 individuals have been reported to have one of two bi‐allelic homozygous missense variants in the *SLC25A42* as the cause of mitochondrial encephalomyopathy, of which 14 of them were of Saudi origin and share the same founder variant, c.871A > G:p.Asn291Asp. The other subject was of German origin with a variant at canonical splice site, c.380 + 2 T > A. Here, we describe the clinical manifestations and the disease course in additional six Saudi patients from four unrelated consanguineous families. While five patients have the Saudi founder p.Asn291Asp variant, one subject has a novel deletion. Functional analyses on fibroblasts obtained from this patient revealed that the deletion causes significant decrease in mitochondrial oxygen consumption and ATP production compared to healthy individuals. Moreover, extracellular acidification rate revealed significantly reduced glycolysis, glycolytic capacity, and glycolytic reserve as compared to control individuals. There were no changes in the mitochondrial DNA (mtDNA) content of patient fibroblasts. Immunoblotting experiments revealed significantly diminished protein expression due to the deletion. In conclusion, we report additional patients with SLC25A42‐associated mitochondrial encephalomyopathy. Our study expands the molecular spectrum of this condition and provides further evidence of mitochondrial dysfunction as a central cause of pathology. We therefore propose that this disorder should be included in the differential diagnosis of any patient with an unexplained motor and speech delay, recurrent encephalopathy with metabolic acidosis, intermittent or persistent dystonia, lactic acidosis, basal ganglia lesions and, especially, of Arab ethnicity. Finally, deep brain stimulation should be considered in the management of patients with life altering dystonia.

**Synopsis**

The study reports a novel deletion that impairs the functional capabilities of *SLC25A42* in patients of Arabian descent.

## 1 INTRODUCTION

Genes encoding membrane proteins are believed to be in large number and consist of significant part of human and mouse genomes.^1^ One of the important membrane molecules is solute carriers (SLCs) that involve in transporting substrates and molecules to their destinations in the living cells. Among SLCs, a subgroup known as SLC25 is critical for substrate transportation into mitochondria and hence indirectly control critical energy processes.^2^, ^3^, ^4^ In human, coenzyme A (CoA) is an essential component for fatty acid and pyruvate oxidation in the cell and biosynthesized outside the mitochondrial matrix; hence, it needs to be transported into mitochondria. *SLC25A42*, a member of as SLC25, is known for its role in the transportation of CoA. Interestingly, this carrier has also capacity to carry adenosine 3′, 5′‐diphosphate perhaps for a role in counter‐exchange transportation of intramitochondrial substrates.^5^ Recently, a single nucleotide substitution in *SLC25A42* was associated with an autosomal recessive human disease characterized by muscle weakness and lactic acidosis.^6^ This missense variant (NM_178526:c.871A > G:p.Asn291Asp) was identified in a single patient from a consanguineous Saudi family.^6^ Subsequently, 12 additional individuals were also identified with the same founder variant. The reported features included hypotonia, developmental delay, intellectual disability, mitochondrial encephalomyopathy, epilepsy, dystonia, elevated serum lactic acid level, and basal ganglia lesions.^7^ Recently, two additional individuals of Saudi and German ethnicity were reported with one harbouring the previously reported founder variant while the latter had a *SLC25A42* splice site variant.^8^

Here, we report six additional affected individuals from four unrelated consanguineous Saudi families, carrying two different *SLC25A42* variants, with five patients harboring the Saudi founder variant and one with a novel deletion.

## 2 METHODS

**FIGURE 1:**
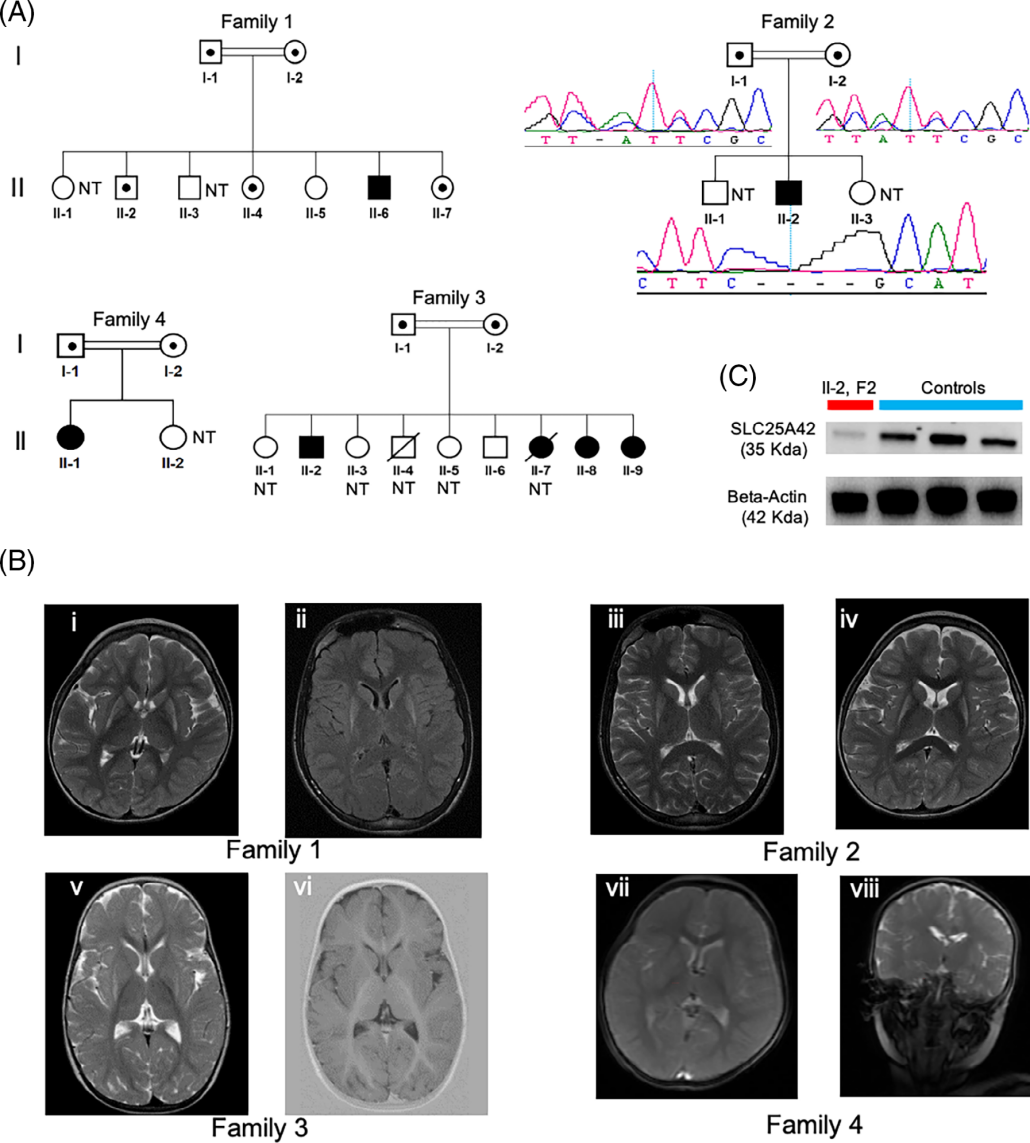
Genetic analysis results. A, Pedigrees of the four families in the study and family segregation analysis for the novel deletion in family 2. Affected individuals are labeled with black colored (filled) symbols. Carriers are represented with half‐filled symbols. In family 2, the Sanger sequencing chromatograms below each individual show the status of each individual. B, Brain MRI pictures of the four index cases from each family. Family 1: Axial T2 (i) and axial FLAIR (ii) images show bilateral symmetrical atrophy and hyperintense signal of the posterior aspect of the putamina. Family 2: (iii, iv) Axial T2‐weighted images show volume loss and hyperintense signal of the bilateral putamina. Family 3: Axial T2 (v) and inversion recovery (vi) show hyperintense T2 signal of the posterolateral aspect of the putamina bilaterally with corresponding hypointense signal on inversion recovery sequence. The putamina have preserved volume. Family 4: There is interval development T2/FLAIR high signal at lateral aspect of both putamen mainly posteriorly with faint T1 low signal and no significant volume loss. No diffusion restriction and no blood degradation product noted. Interval decrease of the prominence of the pericerebral CSF spaces and lateral ventricles was noted. C, Immunoblotting results. The blotting experiments displays significantly reduced protein level in the patient's extracts from cultured fibroblasts in comparison to those of the controls. A polyclonal antibody was targeted against the protein and beta‐actin was used as a control‐loading marker

The study was conducted at King Faisal Specialist Hospital and Research Center. The patients were ascertained after their parents or legal representatives signed a written informed consent form under an institutional review board approved protocol (KFSHRC RAC#2060035 and RAC # 2120022) (Figure 1A).

### 2.1 Sample collection

Blood and skin biopsy collections, and fibroblast cultures were performed according to standard protocols. DNA, RNA, and protein extraction were performed using QIAGEN kits (Venlo, the Netherlands). Nucleic acid quality and quantities were determined via Bioanalyzer 1200 (Agilent Technologies, Santa Clara, California). Proteins were measured by Qubit 3 Fluorometer (Cat# Q33216; Thermo Fisher Scientific). Cell passaging and harvesting were done according to standard protocols.

### 2.2 Affymetrix axiom human mapping assays and autozygosity detection

High‐density axiom arrays (Affymetrix Inc., Santa Clara, California) were utilized for genome‐wide SNP genotyping. Assays were run on Gene Titan (Affymetrix Inc.). Chip hybridization, scanning, image processing, and preliminary data analysis were all done according to manufacturers' protocols and guidelines. Autozygome analysis was performed using AutoSNPa software.^9^, ^10^, ^11^, ^12^, ^13^

### 2.3 Sanger sequencing

DNA was amplified using *SLC25A42* specific primers in separate PCRs. Then each fragment was sequenced using either forward or reversed primers tagged with universal M13 primers according to standard protocols. The sequencing primer used for the c.523_526delCATC were 5’‐GGCTGAAGCAGGAGAATCAC‐3′ (forward) and 5’‐ACCAGCCAGAGTTCCTCTCA‐3′ (reverse) and for the c.871A > G were 5’‐CTCGCCCTGCAGAGTACA‐3′ (forward) and 5′‐ AACACCTCCGGCAGACAG‐3′ (reverse).

### 2.4 Whole exome sequencing and variant filtering

Whole exome sequencing (WES) was undertaken using an Illumina 2500 platform. Libraries were prepared by SureSelect kit (Illumina Inc., San Diego, California). Captured sequences were read on the system and aligned using in‐house pipeline. Generated data were analyzed using publicly and commercially available tools and software. After the analysis of the WES data, the variants were filtered as previously described.^9^, ^10^, ^12^, ^13^, ^14^

### 2.5 mtDNA sequencing

Genomic DNA was extracted from fibroblasts and was used as a template for mtDNA amplification (http://www.mitomap.org/MITOMAP; http://www.mtdb.igp.uu.se/). Complete genome amplification, sequencing, and analyses of sequencing results were all done as detailed elsewhere.^15^, ^16^

### 2.6 Quantitative PCR‐based mtDNA copy number assessment

A quantitative assessment of mtDNA copy number was carried out as previously published.^9^ Briefly genomic DNA from fibroblasts containing mtDNA was used as starting material. *NALCN* and *ND1* were used as nuclear and mitochondrial genome marker genes. Delta delta CT method was utilized for copy number calculation.

### 2.7 XF metabolic assays

We used Seahorse XFp analyzer (Seahorse‐Agilent) to measure oxygen consumption rate (OCR), extracellular acidification rate (ECAR), and cell energy phenotype to determine key parameters of cellular metabolism using control and patient cells as described.^17^, ^18^ One day prior to assay, fibroblasts (20  000 cells/well) were seeded in the Seahorse 8‐well mini culture plate with growth medium (MEM supplemented with 10% FBS, 1% penicillin‐streptomycin, and 1% l‐Glutamine) and placed in 37°C incubator with 5% CO_2_ and allowed to adhere overnight. The sensor cartridge was hydrated in XF calibrant at 37°C in a non‐CO_2_ incubator overnight. Next day, cells were washed twice with warm assay medium, 180 μL of the assay medium was added per well, and the cell culture mini‐plate was placed into a 37°C non‐CO_2_ incubator for 45 to 60 minutes prior to assay.

For the glycolysis stress assay, final concentrations of 10 mM of glucose, 1 μM oligomycin, and 50 mM 2‐deoxyglucose (2‐DG) were used after preparation in the XF base assay medium (supplemented only with 2 mM glutamine [pH 7.4]). While in the mito stress assay, final concentrations of 1 μM oligomycin, 1 μM FCCP, and 0.5 μM Rotenone/antimycin A (Rot/AA) were used after preparation in the XF base assay medium (supplemented with 1 mM pyruvate, 2 mM glutamine, and 10 mM glucose [pH 7.4]). Lastly, the stressor mix of oligomycin and FCCP compounds was combined together to be used in the energy phenotype assay. This mix was prepared by diluting 10 μM oligomycin and 1 μM FCCP (final well concentration) in the XF base assay medium (supplemented with 1 mM pyruvate, 2 mM glutamine, and 10 mM glucose [pH 7.4]). Each individual experiment was done in three replicate wells from control and patient fibroblasts. Results were calculated and produced using the Seahorse Wave software version 2.6.

### 2.8 Immunoblotting

Total cell lysates were obtained from cultured fibroblasts for both patients and controls. To harvest cells, they were allowed to reach confluence, washed with 1x PBS followed by incubation in sufficient amount of trypsin, and briefly incubated in 37°C. Cells were washed again with 1x PBS and then lysed by complete lysis‐M (EDTA‐free) buffer (Roche, Sigma‐Aldrich) according to manufacturers' protocols. Twenty μg/μL of protein and 5 μL of sample buffer containing 2% β‐Mercaptoethanol were mixed. Lysis buffer was added to a final volume of 10 μL. In addition, 5 μL of sample buffer were added to 5 μL of Kaleidoscope prestained standard. Protein transfer was carried out using a Polyvinylidene fluoride membrane (ThermoScientific) previously blocked in 5% skimmed milk in Tris buffered‐saline (TBS) Tween 20 (TBST) prior to immunodecoration with a rabbit polyclonal anti‐SLC25A42 Ab (1:1000; Origene Cat#TA334622) and rabbit β‐actin Ab (1:10 000; Abcam Cat#ab8227). Since both of the antibodies detect proteins of a similar size, the membrane was stripped before β‐actin immunodecoration. Proteins were detected using enhanced chemiluminescence substrate (ThermoScientific). Western blotting experiments were performed in triplicate.

## 3 RESULTS

### 3.1 Clinical descriptions

Brief clinical features of the patients, their pedigree numbers, and patients' codes are listed in Table 1.

**TABLE 1:** Clinical, neurological, and radiological findings of the patients with the *SLC25A42* defect

Patient codes	1	2	3	4	5	6
Family	1	2	3	3	3	4
Pedigree code	II‐6	II‐2	II‐2	II‐8	II‐9	II‐1
Age (years)	12	21	21	10	5	4
Gender	Male	Male	Male	Female	Female	Female
Age at presentation (months)	14	7	10	9	6	14
Birth weight (kg)	3.2	3.0	2.5	2.0	4.0	2.9
Family history	Negative	Negative	Positive	Positive	Positive	Positive of PKU no similar disease
Relatedness of the parents	First cousins	Second cousins	First cousins	First cousins	First cousins	Same tribe
Whole exome sequencing result	*SLC2SA42*: NM_178526.3 (Ch19:19221599G: c.871A > G: p.Asn291Asp. Homozygous. Previously identified Arab founder variant	*SLC2SA42*: NM_178526.3:c.522_524 delCATC: p.173Phe_175ArgPheSer. Homozygous	ND	*SLC25A42*: NM_178526.3:Ch19:19221599:G: c.871A > G: p.Asn291Asp. Homozygous. Previously identified Arab founder variant	ND	1. *SLC25A42*: NM_178526.3:Ch19:19221599:G: c.871A > G: p.Asn291Asp. Homozygous. Previously identified Arab founder variant
Infantile hypotonia	Yes	Yes	Yes	Yes	Yes	Yes
Swallowing/feeding difficulties	Yes	No	Yes	Yes	NGT	Yes
Growth	Below third percentile	Below third percentile	Below third percentile	Below third percentile	Below third percentile	Normal
Weight (kg)	20 (−4.76 *SD*)	45 (−3.45 *SD*)	ND	21 (−2.44 *SD*)	ND	15 (−0.32 *SD*)
Height (cm)	132 (−2.2 *SD*)	153 (−3.4 *SD*)	ND	120 (−2.59 *SD*)	ND	100 (−0.53 *SD*)
Head circumference (cm)	48.8	54.5	ND	51	ND	48
BMI (kg/m^2^)	11.50	19	ND	14.7	ND	15.4
Vision	Normal	Normal	Normal	Normal	Normal	Normal
Hearing	Normal	Normal	Normal	Normal	Normal	Normal
Sleeping	Normal	Normal	Normal	Normal	Abnormal	Normal
Dysmorphism	No	No	No	No	No	No
Period of initial normal milestones	Yes	Yes	No	No	No	Yes
Acute encephalopathy episode at initial presentation	Yes	Yes	No	No	No	Yes
Hospital admission	2 ICU/3 inpatient ward	Few	Few	Few	Few	Frequent
Infantile and childhood motor delay	Yes	Yes	Yes	Yes	Yes	Yes
Involuntary dystonic movement and posturing	Yes	Yes	Yes	Yes	Yes	Yes
Speech delay	Severe	Severe	Severe	Severe	Severe	Severe
Gross receptive language and social interaction	Normal	Normal	Normal	Normal	Normal	Normal
Seizure	Yes	Yes	No	Staring spells but no seizures	Yes	ND
Hyperactivity	No	No	No	No	No	No
Functional status	Wheelchair	Four wheel walker	Ataxia and frequent fall	Wheelchair	Ataxia and frequent fall	Fully mobile
CBC, blood and urine chemistry	Normal	Normal	ND	Normal	ND	Normal
Ammonia (NR 0‐55 umol/L)	44,22	65	ND	Normal	ND	32
Lactic acid (NR 0.5‐2.0) mmol	2.4‐6.9	1.3‐3.4	ND	3.3‐4.2	ND	1.66‐1.89
Acylcarnitine profil	Slightly elevated C10:1‐,C10‐ and C18:1‐carnitine	Normal	ND	Normal	ND	Normal
Urine organic acids	Slight elevation of 3‐hydroxybutyric acid, acetoacetic acid	Normal	ND	Slightly elevated lactate & 3‐hydroxyisovaleric	ND	ND
PDH, PC, PE, and PE testing on fibroblast	Normal	ND	ND	ND	ND	ND
ETC chain complex on the skin fibroblasts	Slightly reduced citrate synthase activity, NADH dehydrogenase (complex1), NADH cytochrome C reductase (complex 1 and 3), succinate dehydrogenase (complex 2), succinate cytochrome C reductase (complex 2 and 3), cytochrome C oxidase (complex 4) were all within normal limits	ND	ND	ND	ND	ND
PDH, PC and PEPCK	Normal	ND	ND	ND	ND	ND
Skeletal survey	Moderate Coxa valga Mild hypoplasia of acetabuli bilaterally	ND	ND	ND	ND	ND
Brain MRI	Axial T2 (i) and axial FLAIR (ii) images show bilateral symmetrical atrophy and hyperintense signal of the posterior aspect of the putamina.. In the MRS study there is no definite lactate peak	Axial T2 weighted images show volume loss and hyperintense signal of the bilateral putamina with MR spectroscopic evidence of lactate within the lesion area	ND	Axial T2 (v) and inversion recovery (vi) show hyperintense T2 signal of the posterolateral aspect of the putamina bilaterally with corresponding hypointense signal on inversion recovery sequence	ND	T2/FLAIR high signal at lateral aspect of both putamen mainly posteriorly with faint T1 low signal and no significant volume loss
EEG	ND	ND	ND	Normal	ND	Normal
Muscle biopsy testing	ND	Minimal thickening of capillaries in the majority of fascicles. Oxidative enzymes showed a slight accentuation of mitochondria with a subsarcolemmal localization. There was no obvious evidence of cytochrome c oxidase deficiency, storage or structural abnormalities in any muscle fibers, although a minimal increase in oxidative enzyme reactivity in the smooth muscles and vessels was noted	ND	ND	ND	ND
Skin biopsy findings	ND	ND	ND	Normal, no specific ultra‐structural deposits observed	ND	ND

Patient 1 (II‐6, family 1) is a 12‐year‐old boy, product of full‐term normal pregnancy with a birth weight of 3.2 kg. The parents are first cousins. All siblings are healthy. Delayed motor and speech development were noted during infancy. He was able to sit with support but not independently and able to roll over from side to side but unable to crawl. He had no meaningful verbal output, but remained alert, interactive with normal vision and hearing. At 14 months of age, and following a mild gastroenteritis, he presented with lethargy, somnolence, abnormal dystonic posturing of the upper limbs, and rapid breathing. He was found to have acute encephalopathy with severe lactic acidosis that required vigorous intravenous hydration and bicarbonate therapy in intensive care. At 20 months of age, he had a similar episode but less severe. Following these episodes, his condition has remained static with almost no further progress in his milestones. Brain magnetic resonance imaging (MRI) revealed bilateral symmetrical basal ganglia T2 hyperintensity with diffusion restriction seen in the caudate and the lentiform nuclei. At the most recent physical examination, his growth parameters are below third percentile for age. He is wheelchair bound, has prominent central hypotonia, and dystonic posturing when he attempts to reach for objects. He has no expressive language but remains alert and interactive. Investigations showed high serum lactate on two occasions at 6.9 mmol/L and 5.1 mmol/L (normal range, 0.7‐2.1 mmol/L). Urine organic acid reveled slight elevation of 3‐hydroxybutyric acid, acetoacetic acid, and lactic acid. Enzymatic activities of pyruvate dehydrogenase, pyruvate carboxylase, and phosphoenolpyruvate carboxykinase in fibroblasts were normal. Mitochondrial respiratory chain studies in fibroblasts revealed a slightly decreased citrate synthase activity but otherwise normal (data not shown).

Patient 2 (II‐2, family 2) is a 21‐year‐old male, product of a full‐term normal pregnancy with a birth weight of 3.0 kg. His parents are healthy second cousin. At 7 months of age, he developed acute symptoms of lethargy, somnolence, and abnormal dystonic posturing of the upper limbs with rapid breathing associated with severe lactic acidosis. He was managed in intensive care with vigorous hydration and bicarbonate therapy. At 2 years of age, he was able to sit without support and started to walk with assistance but with some dystonic movements. His speech was delayed but had normal vision and hearing. He continued to have recurrent metabolic crises and later developed seizures that were controlled with antiepileptic medications. Current examination revealed growth parameters below third percentile for age. He has prominent central hypotonia and dystonic posturing of neck and both upper and lower extremities. Serum lactate was mildly elevated on two occasions at 2.2 and 3.4 mmol/L (normal range 0.7‐2.1 mmol/L) with subsequent normal levels. Brain MRI at age of four, showed slight atrophy of the posterior parts of the putamena bilaterally in conjunction with signal abnormalities both on the T1‐ and the T2‐weighted images. Magnetic resonance spectroscopy (MRS) showed a slight decrease in the creatine peak with respect to the choline in conjunction with small amount of lactate. With these findings, a genetic mitochondrial disease was suspected including Leigh's disease. Mitochondrial DNA (mtDNA) and *SLC19A3* sequencing for biotin‐responsive basal ganglia disease (BBGD; OMIM 607483) were unremarkable. Muscle biopsy revealed minimal thickening of capillaries in the majority of fascicles. Oxidative enzymes showed a slight accentuation of mitochondria with a subsarcolemmal localization. There was no evidence of cytochrome *c* oxidase deficiency, storage, or structural abnormalities in any muscle fibers, although a minimal increase in oxidative enzyme reactivity in the smooth muscles and vessels was noted. At the age of 17 years, he underwent deep brain stimulation (DBS) at the globus pallidus internus bilaterally following which, he showed significant improvement in his dystonic posturing, balance, and motor function enabling him to get out of wheelchair and use four‐wheel walker instead.

Patient 3 (II‐2, family 3) (only limited clinical information is available) is a 21‐year‐old male born normally with a birth weight of 2.5 kg. He is the older sibling of patient 4. At the age of 10 months, he was found to have developmental delay with central and peripheral hypotonia. He was not able to sit without support, stand, or walk. Dystonic movements and posturing of the upper extremities were noted at times. At the age of 6 years, his mother reported improvement in developmental skills. The patient was able to stand without support, take one to two steps, crawl slowly, and feed himself. He was able to say baba, mama, and comprehend simple commands. At age seven, he started walking with unsteady gait. His hearing and vision have remained normal. At the age of 19 years, he attended the school with acceptable performance.

Patient 4 (II‐8, family 3) is a 10‐year‐old girl, product of full‐term caesarian section delivery due to failure to progress and fetal distress. Birth weight was 2 kg with good APGAR score. At the age of 9 months, she presented with history of floppiness and frequent staring spells lasting for few seconds. No abnormal movements of the extremities were reported. She had no head control and was not able to sit. On examination, her growth parameters were below third percentile. She had prominent central hypotonia with dystonic posturing. Lactic acid level ranged from normal value of 1.4 to mildly elevated level of 4.2 mmol/L. Acylcarnitine profile and plasma amino acids were normal apart from mildly elevated alanine. Urine organic acids revealed elevated lactate. electroencephalogram (EEG) was normal. Brain MRI at age of 13 months was normal. At the age of three, her mother reported improvement in the development. The patient was able to stand without support, take one to two steps, crawl slowly, and feed herself. She was able to say baba, mama, and comprehend simple commands. At the age of 6 years, she showed further improvement in motor skills as she started walking with unsteady gait and mild dystonia.

Patient 5 (II‐9, family 3) (only limited clinical information is available) is a 5‐year‐old female and the younger sibling of patients 3 and 4. She is a product of full‐term lower segment caesarian section (LSCS) delivery due to large size of 4 kg, secondary to maternal gestational diabetes. At 6 months of age, she was noted to have central and peripheral hypotonia associated with failure to thrive. Her growth parameters are below third percentile for age. She is bed bound and fed via nasogastric tube. She has dystonic posturing with abnormal movements of the upper extremities. She has required several hospital admissions due to feeding problems with frequent vomiting, choking, difficulty in breathing, recurrent fever associated with seizures, and aspiration pneumonia.

Patient 6 (II‐2, family 4) is a 4‐year‐old girl, born normally with a birth weight of 2.9 kg. She had normal acquisition of milestones during infancy. At 14 months of age, she presented with fever, severe lethargy, somnolence, and abnormal dystonic posturing of the upper limbs with rapid breathing. She was found to have acute encephalopathy, severe lactic acidosis, and hyperammonemia requiring intensive care management. This was followed by loss of her motor milestones with dystonic posturing of the neck and upper extremities. Her growth parameters including head circumference were normal. No dysmorphic facial features were noted. Head and neck, chest, cardiovascular, and abdominal examination were unremarkable. Central nervous system (CNS) examination revealed central hypotonia with intermittent dystonic movement of the arms, trunk, and lower extremities. MRI of the brain of T2/FLAIR showed high signal at lateral aspect of both putamen mainly posteriorly. She had no further episodes of metabolic decompensation and her latest serum lactate level was normal.

## 4 GENETICS AND MOLECULAR STUDIES

We first performed mtDNA sequencing of the collected samples that revealed no pathogenetic variants. We then focused on likely pathogenic variants in nuclear genes. As part of a routine workflow, we performed autozygosity mapping based on genome‐wide SNP calls to detect shared runs of homozygosity (ROHs) in the family (family 1) as well as WES in the index case. Based on established protocols, we proceeded to iterative filtering as described previously.^9^, ^10^, ^11^, ^12^, ^13^ The analysis yielded the previously reported, single homozygous variant in *SLC25A42* (NM_178526.3:c.871A > G:p.Asn291Asp) in patient 1^6^ (Figure S1). The variant was fully segregated in the family 1 with the exception of a boy and a girl who were not tested due to unavailability of their DNA samples (Figure 1A). During the course of these experimental procedures, we were able to identify additional five individuals with similar clinical features and presentation from three other families (Figure 1A and Table 1). Although these families were unrelated, the three of them were from the same tribe. Hence, we proposed the *SLC25A42* may harbor the culprit variants due to the presence of myopathy in all the patients. Interestingly, our approach revealed a novel deletion (leading to truncation) in *SLC25A42* (NM_178526.3; c.523_526delATCC; p.Ile175Alafs*8) in patient 2 and the founder variant (p.Asn291Asp) in the remaining patients in families 3 and 4. Segregation analyses revealed that both are fully supplemental figure segregated with the phenotype in the families (Figure 1A, S2).

To gain a functional insight into the likely consequence of protein truncation due to the deletion, we performed immunoblotting experiments using the patient's fibroblasts. Our results revealed a marked loss of SLC25A42 steady‐state levels in the fibroblasts from the patient (Figure 1C).

**FIGURE 2:**
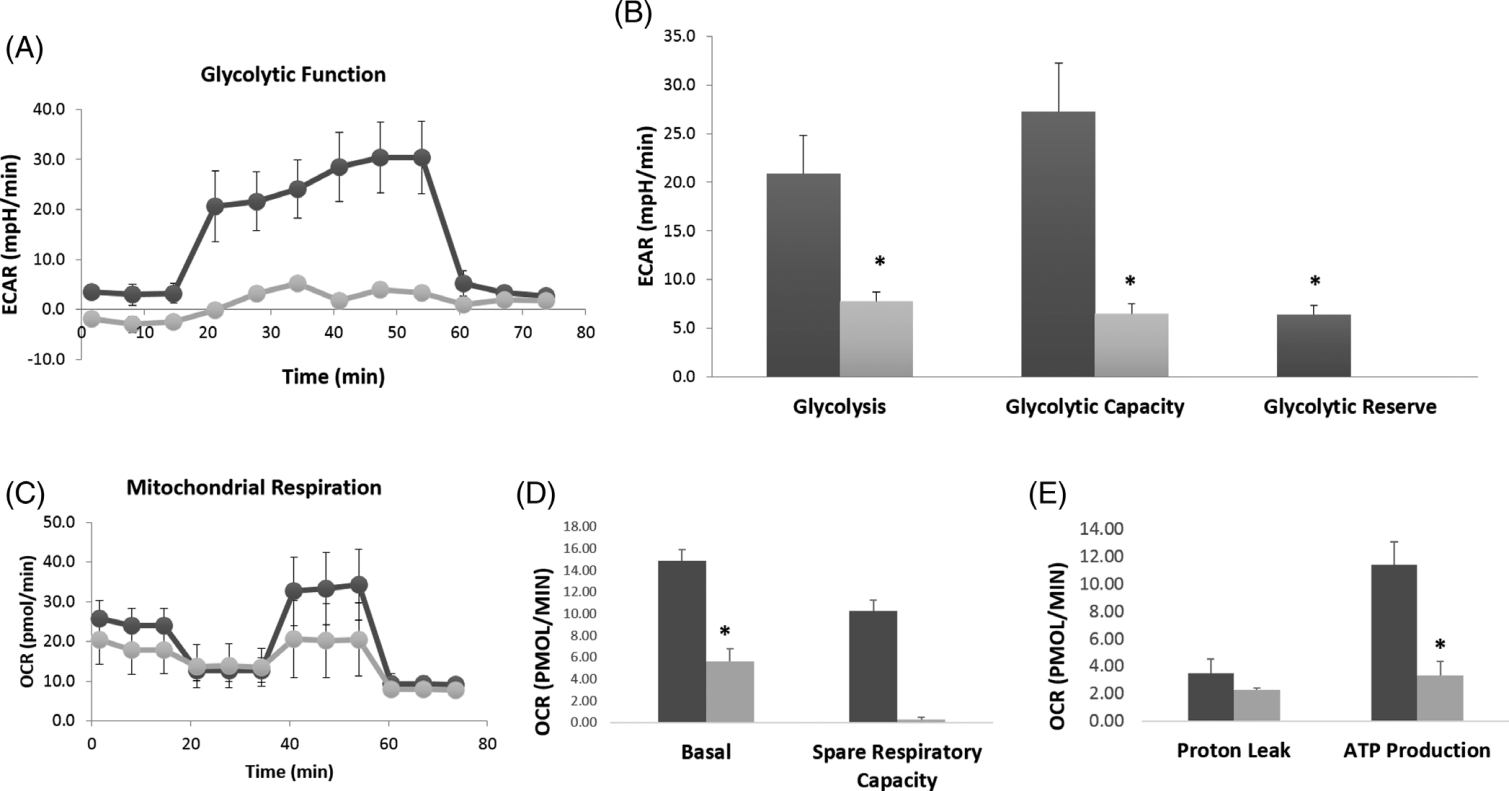
The XF metabolic assays. The ECAR and OCR were measured in fibroblasts from control subject and patient fibroblasts. Both glycolytic functions (Figure 2A,B) and mitochondrial functions (Figure 2C–E) were significantly reduced in the patient fibroblasts as compared to control. Data are presented as mean ± SEM. Each treatment was measured every 6.5 minutes for three times and three replicates were utilized per condition. *P < .05 control vs affected individuals' cells (two‐tailed Student's *t*‐test); black for control, light gray for affected individual

To further investigate the cellular and organeller consequences of SLC25A42 loss, we performed a glycolysis stress test on the fibroblasts (patient 2 vs controls), which revealed significantly lower glycolytic function in the patient cells. Moreover, ECARs for glycolysis and glycolytic capacity in the patient cells were significantly lower than those in the control cells (*P*‐value < .05) (Figure 2A,B). We then proceeded to check mitochondrial respiration parameters in the same cells. A similar pattern of stress profile was observed in the cells. The patient's cells showed a decrease in all mitochondrial respiration parameters as compared to those of the control (Figure 2C‐E). Indeed, the OCRs for basal, spare‐respiratory capacity, and ATP production were significantly decreased in the affected individual compared to those of the control cells (*P* < .05) (Figure 2C‐E). We also evaluated cell energetic phenotyping that revealed no big difference between the cells from the patient and controls as both responded similarly at stressed conditions in cell energy phenotype and metabolic potential.

## 5 DISCUSSION

A bi‐allelic deleterious (c .871A > G, p.Asn291Asp) *SLC25A42* variant was first described as a cause of mitochondrial myopathy in a 16‐year‐old boy born to consanguineous Saudi parents.^6^ Later, a follow‐up study by the same group reported 12 additional individuals with the same founder variant and provided more detailed description of the phenotypic spectrum of the disease. The authors highlighted the wide clinical heterogeneity even within the same family and the lack of meaningful response to treatment with biotin, thiamine, riboflavin, and coenzyme Q10.^7^ Recently, another study reported the founder variant in another Saudi patient and a novel splicing variant c.380 + 2 T > A in a German patient, the first ethnically different individuals affected with the disease.^8^ The German patient had similar disease characteristics seen in some of the reported Saudi cases such as developmental delay, milder version of ID, muscular hypotonia, and the brain MRI findings of abnormal bilateral signal alterations in the caudate nucleus. Hence, to this date only two homozygous variants (a missense variant: p.Asn291Asp, and a canonical splice site variant: c.380 + 2 T > A, leading to a splicing error) in a total of 15 patients have been identified, of which 14 subjects have the missense founder variant and all are from Saudi Arabia.

In this study, we report six additional patients with pathogenic *SLC25A42* variants and compared them to the previously reported patients. There is a wide clinical heterogeneity in the presentation of the patients from near normal to severe motor and speech impairment. Patients can present with an initial normal or near normal development followed by acute metabolic decompensation in infancy or childhood, characterized by lethargy, somnolence, encephalopathy, abnormal dystonic posturing of the upper limbs, rapid breathing, and moderate‐to‐severe metabolic acidosis. The metabolic crisis often requires intensive care admission, but the acute illness recovers with vigorous hydration and attention to acidosis. Some patients present with hypotonia and motor developmental delay from the beginning without an acute decompensation. Recovery following first metabolic decompensation is characterized by a variable period of loss of previously acquired milestones, followed by static development and subsequent slow recovery of some motor milestones. Speech is severely affected in the majority of the patients; however, receptive language and social skills were well developed in our cohort. Patients remain socially interactive and understand commands well. Movement disorder is a common observation in many metabolic disorders associated with energy deficiency.^19^ Indeed, in our cohort, all patients had abnormal dystonic movement and posturing. The dystonia started after the first metabolic crisis and was noted to be much more pronounced during acute episodes. This is also consistent with MRI brain findings of basal ganglia involvement. Four patients with MRI brain (1, 2, 3, and 6) had bilateral putamen involvement and especially the posterior part. This is similar to five patients in the previous reports.^7^, ^8^

The only consistent finding in the metabolic work up is elevated serum lactate especially during the crisis. In between the crisis, lactate is often normal or mildly elevated. We did not observe any clinical benefit with biotin, thiamine, riboflavin and coenzyme Q10, which is consistent with the previous observation.^7^

DBS involves implanting electrodes within the brain and has been reported to be modestly effective in treating rare inherited dystonias with a combined phenotype.^20^ DBS was also reported to be used in a 4‐year‐old boy with methylmalonic acidemia with total body dystonia with a marked improvement and reduction in pain.^21^ Our oldest patient 2 (II‐2) had severe progressive dystonia causing significant limitation of his movements and dependency on wheel chair. With the above encouraging reports, our patient underwent DBS at the globus pallidus internus bilaterally at 17 years. A significant improvement was noted in his balance and motor function. Dystonic posturing and movement improved, enabling him to sit and walk more comfortably using a four wheeler.

Western blotting results using patient 2's fibroblasts confirmed a marked reduction in steady‐state levels of immunoreactive SLC25A42, providing additional evidence of pathogenicity for this novel deletion. Regardless of the exact mechanism, this variant may affect protein synthesis or stability making some functions of the SLC25A42 to be comprised being a loss‐of‐function variant. Also, the loss of SLC25A42 could be explained by impaired transportation of CoA and other molecules to the mitochondria.^5^

Moreover, we assessed the mitochondrial functions likely to be affected by the novel variant. Both glycolytic stress and mitochondrial stress assays showed decrease in the glycolytic and mitochondrial parameters in the patient 2'cells as compared to those of the control. Also, the quantitative PCR showed no changes in the mtDNA content of the patient's fibroblasts. This indicates that the energetic defect in patient cell was due to mitochondrial dysfunction not to mtDNA content.

Since the mitochondria and cellular energy production are under the control of both mitochondrial and nuclear encoded genes,^22^, ^23^, ^24^ it is possible that reduction in both OCR and ECAR could be attributed to biochemical changes, which may directly or indirectly interrupt the nuclear and mitochondrial communication resulting in different gene expression affecting the cellular function and metabolism.^25^, ^26^ CoA is known to be one of the essential coenzymes in the citric acid cycle.^27^ It has important roles in the synthesis and oxidation of fatty acids, ketogenesis, biosynthesis of cholesterol and acetylcholine, regulation of gene expression, and cellular metabolism.^28^, ^29^, ^30^ In addition, CoA functions are critical for early metabolic pathways^31^, ^32^ and for the development of the nervous system.^33^ Defects in the transportation of acetyl‐CoA to the mitochondria would result in the accumulation of the enzyme in the cytoplasm. This accumulation has shown to compromise protein function, metabolism,^34^ and contributed to inherited metabolic disorders.^35^, ^36^ Moreover, CoA is crucial for acyl carrier proteins which have essential role in mitochondrial functions^5^ and nonribosomal protein biosynthesis.^24^ Giving that the main function of SLC25A42 is the transport of CoA into the mitochondria in exchange for adenine nucleotides,^5^ defects in such transport due to variants in the *SLC25A42* including the one reported in our work would result in impaired metabolic pathways leading to pathogenicity.

In conclusion, we report six additional patients with SLC25A42‐associated mitochondrial encephalomyopathy. We expand the molecular spectrum and provide further evidence of mitochondrial dysfunction as the central cause of pathology. We therefore propose that this disorder should be included in the differential diagnosis of patients (especially for those of Arab ethnicity) with unexplained motor and speech delay, recurrent encephalopathy and metabolic acidosis, intermittent or persistent dystonia, lactic acidosis, and basal ganglia lesions. Finally, DBS should be considered in the management of patients with SLC25A42‐associated mitochondrial encephalomyopathy and life altering dystonia.

## CONFLICT OF INTEREST

The authors have no conflicts of interest to declare.

## AUTHORS CONTRIBUTIONS

Namik Kaya conceived and designed the experiments. Mazhor Aldosary, Shahad Baselm, Maha Abdulrahim, Rawan Al‐Mass, Moeenaldeen D. AlSayed, FAM, Laila AlQuait, Albandary AlBakheet, Basma Alahideb, Tarfa Al‐Shidi, Eman Al‐Obeid, Basma Alahideb, Lujane Alahaideb performed the experiments. Namik Kaya, Mazhor Aldosary, and Dilek Colak analysed the data. Namik Kaya, Mazhor Aldosary, Shahad Baselm, Maha Abdulrahim, Maysoon Alsagob, Moeenaldeen D. AlSayed, Rozeena Huma, Zainab AlMasseri, Robert W. Taylor, Dilek Colak wrote the paper. Moeenaldeen D. AlSayed, Namik Kaya, Dilek Colak, Robert W. Taylo, Zainab AlMasseri and Mazhor Aldosary revised the manuscript. Namik Kaya, Dilek Colak, Robert W. Taylor, Zainab AlMasseri and Mazhor Aldosary involved in analysis and interpretation of the data and revised the writing critically. Moeenaldeen D. AlSayed, Rozeena Huma, Alya Qari, Zainab AlMasseri, Maysoon Alsagob, and Laila AlQuait collected specimen, handled biopsies, undertook patient care and management, collected clinical data, and delineated the patients' phenotype.

## PATIENT CONSENTS

Obtained.

## ETHICS APPROVAL

King Faisal Specialist Hospital and Research Center, Riyadh, Saudi Arabia.

## Supporting information


**Supplemental Figure S1** WES filtering steps of the NM_178526: c.871A > G: p.Asn291Asp variant in family 1. The WES filtering steps present the number of detected variants in each step. The Sanger sequencing analysis identified the deletion (NM_178526.3; c.523_526delATCC; p.Ile175Alafs*8) in family 2. The diagram shows that deletion causes out of frame leading to a truncated premature protein.

**Supplemental Figure S2** Segregation analysis of the p.Asn291Asp variant. Sanger sequencing results indicate full segregation of the variant in the families 1, 3, and 4.
