# Supplementary figures and images for: SLC25A42‐associated mitochondrial encephalomyopathy: Report of additional founder cases and functional characterization of a novel deletion
Source: JIMD Rep. 2021 May 4;60(1):75–87. doi: 10.1002/jmd2.12218 (PMC8260478; doi:10.1002/jmd2.12218)

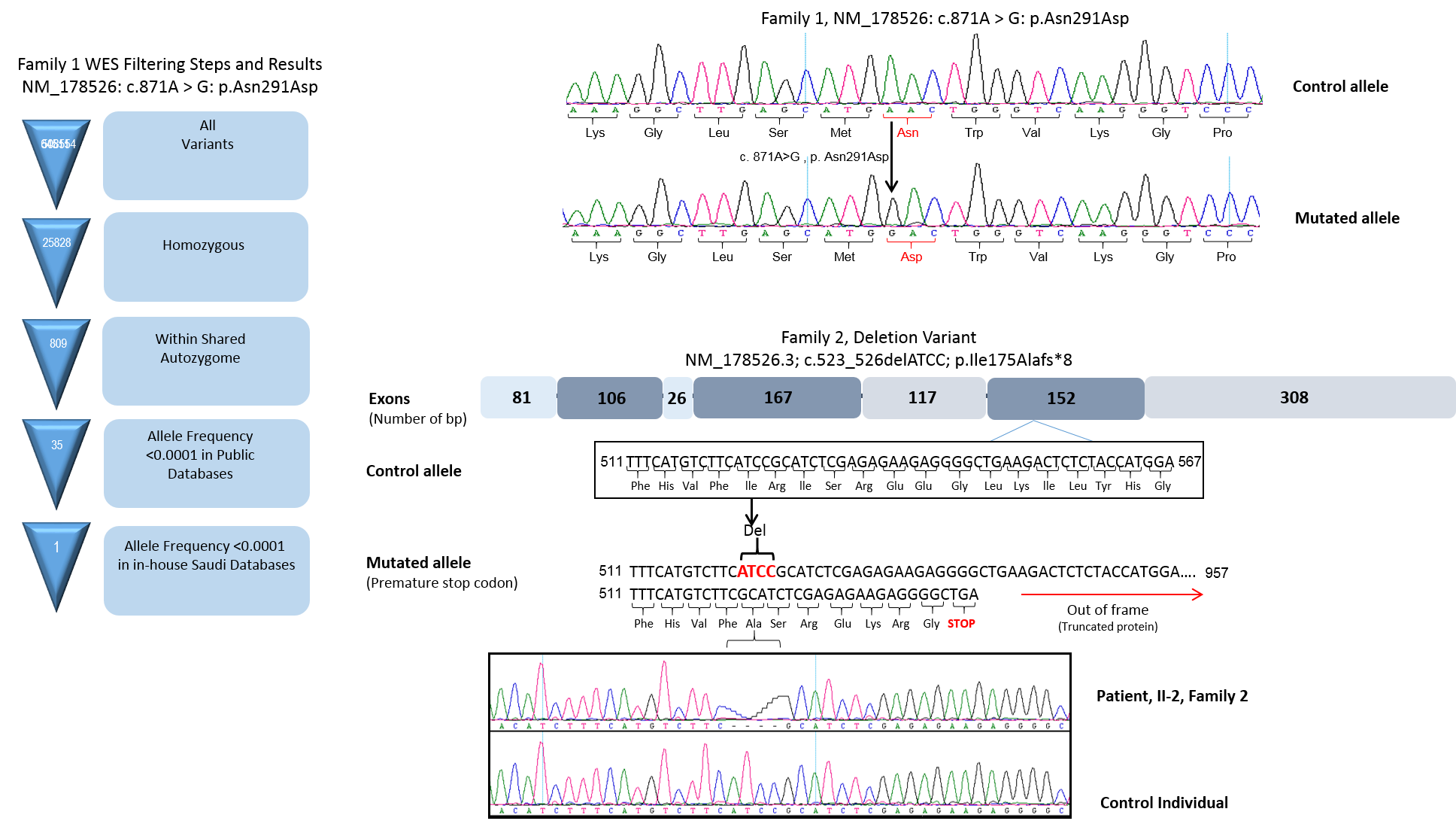

Supplement: Supplementary file 1 — Supplemental Figure S1 WES filtering steps of the NM_178526: c.871A > G: p.Asn291Asp variant in family 1. The WES filtering steps present the number of detected variants in each step. The Sanger sequencing analysis identified the deletion (NM_178526.3; c.523_526delATCC; p.Ile175Alafs*8) in family 2. The diagram shows that deletion causes out of frame leading to a truncated premature protein. [file JMD2-60-75-s001.png]

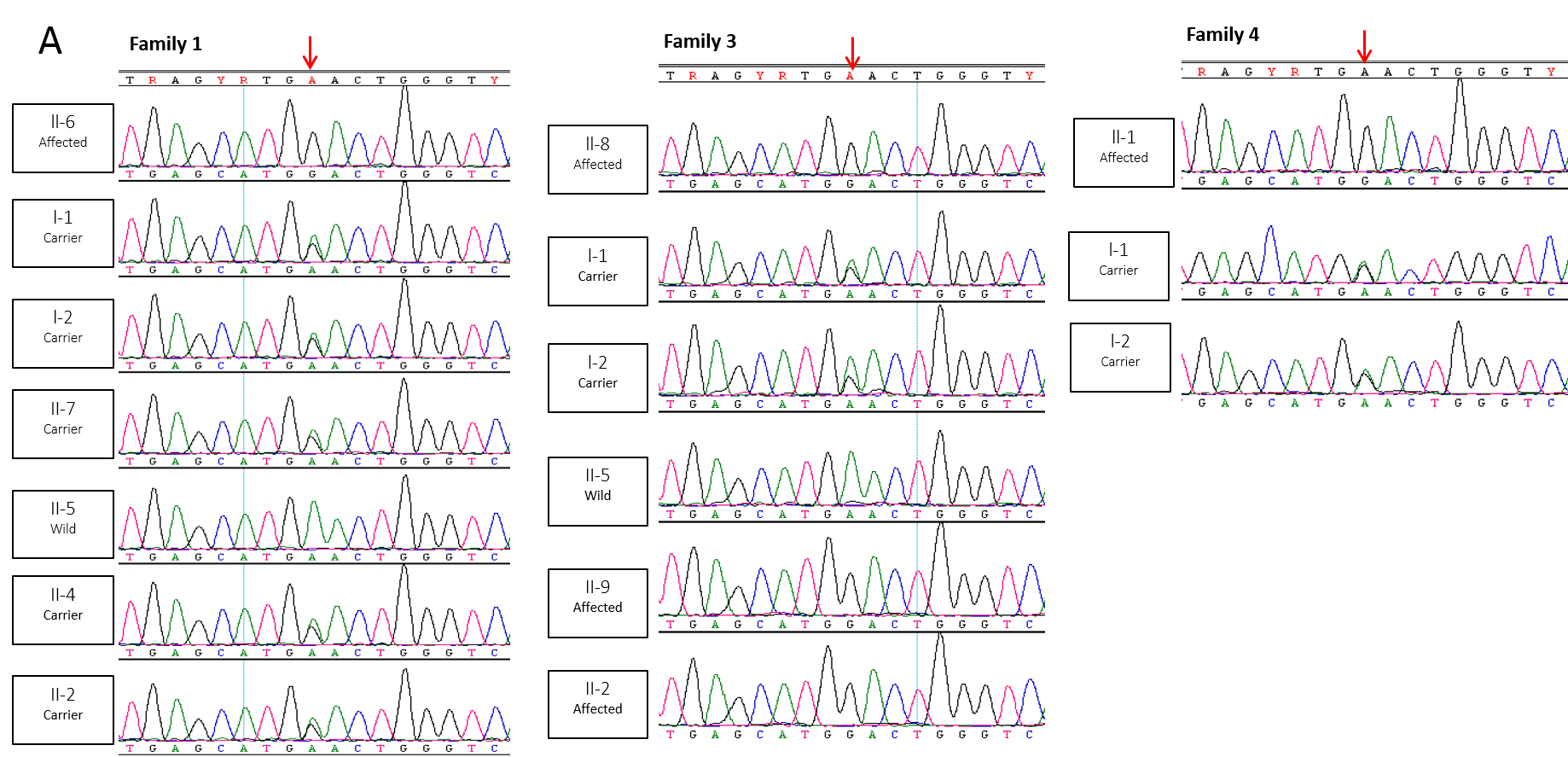

Supplement: Supplementary file 2 — Supplemental Figure S2 Segregation analysis of the p.Asn291Asp variant. Sanger sequencing results indicate full segregation of the variant in the families 1, 3, and 4. [file JMD2-60-75-s002.png]
